# Secretory Defect and Cytotoxicity: THE POTENTIAL DISEASE MECHANISMS FOR THE RETINITIS PIGMENTOSA (RP)-ASSOCIATED INTERPHOTORECEPTOR RETINOID-BINDING PROTEIN (IRBP)*

**DOI:** 10.1074/jbc.M112.418251

**Published:** 2013-03-13

**Authors:** Songhua Li, Zhihui Yang, Jane Hu, William C. Gordon, Nicolas G. Bazan, Arthur L. Haas, Dean Bok, Minghao Jin

**Affiliations:** From the ‡ Department of Ophthalmology and Neuroscience Center and; the ¶ Department of Biochemistry and Molecular Biology, Louisiana State University Health Sciences Center, New Orleans, Louisiana 70112 and; the § Jules Stein Eye Institute and Department of Neurology, David Geffen School of Medicine, UCLA, Los Angeles, California 90095

**Keywords:** ER Stress, Extracellular Matrix, Photoreceptors, Protein Misfolding, Retinal Degeneration, Retinoid-binding Protein

## Abstract

Interphotoreceptor retinoid-binding protein (IRBP) secreted by photoreceptors plays a pivotal role in photoreceptor survival and function. Recently, a D1080N mutation in *IRBP* was found in patients with retinitis pigmentosa, a frequent cause of retinal degeneration. The molecular and cellular bases for pathogenicity of the mutation are unknown. Here, we show that the mutation abolishes secretion of IRBP and results in formation of insoluble high molecular weight complexes via disulfide bonds. Co-expression of protein disulfide isomerase A2 that regulates disulfide bond formation or introduction of double Cys-to-Ala substitutions at positions 304 and 1175 in D1080N IRBP promoted secretion of the mutated IRBP. D1080N IRBP was not transported to the Golgi apparatus, but accumulated in the endoplasmic reticulum (ER), bound with the ER-resident chaperone proteins such as BiP, protein disulfide isomerase, and heat shock proteins. Splicing of X-box-binding protein-1 mRNA, expression of activating transcription factor 4 (ATF4), and cleavage of ATF6 were significantly increased in cells expressing D1080N IRBP. Moreover, D1080N IRBP induced up-regulation and nuclear translocation of the C/EBP homologous protein, a proapoptotic transcription factor associated with the unfolded protein response. These results indicate that loss of normal function (nonsecretion) and gain of cytotoxic function (ER stress) are involved in the disease mechanisms of D1080N IRBP. Chemical chaperones and low temperature, which help proper folding of many mutated proteins, significantly rescued secretion of D1080N IRBP, suggesting that misfolding is the molecular basis for pathogenicity of D1080N substitution and that chemical chaperones are therapeutic candidates for the mutation-caused blinding disease.

## Teaser

**Background:** D1080N mutation in interphotoreceptor retinoid-binding protein (IRBP) causes retinitis pigmentosa with unknown mechanisms.

**Results:** The mutation abolished IRBP secretion and induced endoplasmic reticulum stress by forming insoluble IRBP-containing complexes via disulfide bonds.

**Conclusion:** Loss of normal function and gain of cytotoxic function are the likely mechanisms for retinal degeneration.

**Significance:** Chaperones that prevent misfolding of D1080N IRBP are therapeutic candidates.

## Introduction

Retinitis pigmentosa (RP),^4^ characterized by progressive degeneration of photoreceptors, is a genetically heterogeneous group of inherited retinal diseases affecting 1 in 3000–7000 people (1). Recently, a mutation leading to an Asp-to-Asn substitution at position 1080 (D1080N) in interphotoreceptor retinoid-binding protein (IRBP) was found in patients with autosomal recessive RP (2). The mutated Asp residue is completely conserved in all four homologous modules of vertebrate IRBP and in C-terminal processing proteases found in bacteria, algae, and plants. Patients with the mutation displayed profound loss of rod photoreceptor function and severely diminished cone photoreceptor function (2). The pathogenic mechanism by which D1080N mutation in *IRBP* causes RP remains unknown.

Mammalian IRBP is a 140–145-kDa glycoprotein (3) with four homologous modules each consisting of ∼300 amino acid residues (4, 5). IRBP is secreted by photoreceptors into the interphotoreceptor matrix (6, 7) where it protects and solubilizes the visual cycle retinoids by physically binding with 11-*cis*-retinaldehyde, 11-*cis*-retinol, and all-*trans*-retinol (8, 9). In *ex vivo* experiments, IRBP promotes release of 11-*cis*-retinaldehyde from retinal pigment epithelium (RPE) (10) and all-*trans*-retinoid from the neural retina after photobleaching of rhodopsin (11–13). Consistent with these results, *in vivo* studies demonstrated that IRBP plays an important role in the RPE-dependent and Müller cell-dependent visual cycles (13, 14). Because IRBP is expressed before the visual cycle is operational (15–17), its participation in retinal development has been suggested. Studies on IRBP-deficient mice (18) suggest that differentiation of retinal cells, including cone and rod photoreceptors in *Irbp*^−^*^/^*^−^ mice, was similar to that in wild-type mice (13, 19). However, *Irbp*^−^*^/^*^−^ mice showed an early progressive degeneration of rod and cone photoreceptors (13, 18–20). Dark rearing could not prevent degeneration of photoreceptors in *Irbp*^−/−^ mice (13, 20), indicating that the photoreceptor degeneration is not due to an aberrant visual cycle. These studies suggest that IRBP has an unidentified function that is essential for photoreceptor survival. Because the complete mechanism of IRBP function remains unknown, it is difficult to predict the mechanism by which D1080N IRBP causes RP.

Many mutations associated with retinal degeneration lead to production of misfolded proteins by retinal cells (21). In most cases, secretory or transmembrane proteins that fail to assume their native structure do not transit to the Golgi apparatus. Such misfolded proteins often are subject to ER-associated degradation (ERAD) via retrotransport to the cytosol followed by ubiquitination and proteasomal degradation (22). However, when folding mutants of secretory proteins are not efficiently degraded, they accumulate in the ER, cause ER stress, and trigger the unfolded protein response (UPR). The ER stress-associated UPR is a set of conserved intracellular signaling pathways that involve transcriptional induction of ER-resident chaperones and foldases through activation of several transcription factors, including protease-dependent activation of the activating transcription factors 6 (ATF6), mRNA splicing-dependent production of mature X-box-binding protein-1 (XBP-1), and transcriptional activation of ATF4 (23–26).

The UPR protects cells from the toxic effects of misfolded proteins in the early phase. However, prolonged and/or excessive accumulation of misfolded proteins in the ER activates an UPR-specific apoptotic program, which contributes to the pathogenesis of retinal degeneration associated with ER stress (27–33). One important signal leading to cell apoptosis is the transcriptional induction of the C/EBP homologous protein (CHOP), the proapoptotic transcription factor that switches the adaptive response to cell death pathways (34).

To define the pathogenic mechanism(s) associated with the IRBP mutation causing RP, we investigated the properties of D1080N IRBP with regard to its secretion, subcellular localization, aggregate formation, degradation, interactions with other proteins, ER stress, and rescue. We found that the RP-associated mutation abolished secretion and resulted in protein misfolding, which formed high molecular weight complexes (HMCs) via protein disulfide isomerase (PDI)-modulated disulfide bonds. D1080N IRBP was retained in the ER rather than transported to the Golgi apparatus, inducing ER stress and concomitant up-regulation of UPR-associated proapoptotic CHOP. Treatment of cells with 4-phenylbutyrate (PBA), glycerol, low temperature, and Cys-to-Ala substitutions or overexpression of PDI significantly promoted secretion of the mutant IRBP.

## EXPERIMENTAL PROCEDURES

### 

#### 

##### Cell Culture and Transfection

The mouse photoreceptor-derived 661W cells (35) and 293T-LC cells (36) were maintained in a 5% CO_2_ incubator as described previously. PolyJet (SignaGen Laboratories) or Lipofectamine 2000 transfection reagent (Invitrogen) was used to introduce plasmids into the cells. Transfection efficiency was determined by calculating the ratio of transfected gene-positive cells to DAPI-positive cells in randomly chosen areas (400 × 400 μm) of cell cultures transfected in triplicate. For ER stress assays, the culture media were changed every 6 h after transfection.

##### Low Temperature and Chemical Treatment of Cells

For low temperature treatment, cells expressing wild-type (WT) or D1080N IRBP were incubated at 30 °C or 26 °C for the indicated times. For chemical treatment, cells were incubated with the following reagents at the indicated final concentration in the culture media for the indicated times at 37 °C. Chemical chaperones: 4 mm PBA (Sigma-Aldrich) and 5% glycerol were incubated with cells for 48–96 h; proteasome and E1 inhibitors: 10 μm MG132 (Sigma-Aldrich) and 30 μm PYR-41 (Biogenova) were incubated with cells for 16 h.

##### Plasmids and Site-directed Mutagenesis

The mammalian expression vector pRK5-IRBP (13), which encodes human IRBP, was used as a template to generate mutant IRBP expression plasmids. Point mutations for D1080N and the indicated Cys-to-Ala substitutions were introduced by PCR using a QuikChange II XL Site-directed Mutagenesis Kit (Stratagene). Constructs for wild-type and D1080N IRBPs with a C-terminally fused FLAG or HA epitope were made by PCR using a primer containing the epitope sequence (all primers are listed in Table 1). Following mutagenesis, all sequences of the plasmids were confirmed by DNA sequence analysis. The complete coding regions of the mouse protein disulfide isomerase A2 (GenBank Accession BC116671) or binding immunoglobulin protein (GenBank Accession AJ002387) amplified by PCR were subcloned into the pRK5 vector. Plasmids for mammalian cell transfection were purified using the PureLink HiPure Plasmid DNA Purification Kit (Invitrogen).

**TABLE 1:** **Primers for introducing mutation, FLAG epitope, or HA epitope into IRBP**

Mutation	Primer sequences (F: forward, R: reverse)
D1080N	F: 5′-acggatgccatgatcatcaatatgaggttcaacatcggtg-3′
	R: 5′-caccgatgttgaacctcatattgatgatcatggcatccgt-3′
C40A	F: 5′-catgtccgtgctgctcgctggcctggctggcc-3′
	R: 5′-ggccagccaggccagcgagcagcacggacatg-3′
C172A	F: 5′-gctggatctccggcacgccacaggaggccaggtc-3′
	R: 5′-gacctggcctcctgtggcgtgccggagatccagc-3′
C304A	F: 5′-gcggggtgctgcccgctgtggggactccggcc-3′
	R: 5′-ggccggagtccccacagcgggcagcaccccgc-3′
C797A	F: 5′-ccatcccgctgctcgcctcctacttctttgaggc-3′
	R: 5′-gcctcaaagaagtaggaggcgagcagcgggatgg-3′
C1096A	F: 5′-cctccattcccatcttggcctcctacttctttgatgaag-3′
	R: 5′-cttcatcaaagaagtaggaggccaagatgggaatggagg-3′
C1175A	F: 5′-gtgaccagtgggggcgcccagccaccacagacc-3′
	R: 5′-ggtctgtggtggctgggcgcccccactggtcac-3′
FLAG*^a^*	R: 5′-cctcgagttacttatcgtcgtcatccttgtagtcctgcaggcctgggctc-3′
HA*^a^*	R: 5′-cctcgagctaagcgtagtcaggtacatcgtatgggtacaggtggtcctgcaggcc-3′

*^a^* These primers contain a stop codon after the FLAG or HA epitope and also contain an XhoI site after the epitope sequence. The FLAG or HA epitope was fused to the C terminus of IRBP.

##### Antibodies

A polyclonal antibody against bovine IRBP purified from bovine interphotoreceptor matrix by affinity chromatography on a concanavalin A column was generated in rabbit. The immunoglobulin G (IgG) fraction purified from the rabbit antiserum was used for immunoblotting and immunostaining. Other primary antibodies used are specific for the following proteins or peptides: ATF4 from Santa Cruz Biotechnology; ATF6, KDEL, and 58K-Golgi from Abcam Inc.; CHOP, BiP, and FLAG tag from Cell Signaling Technology; HA tag and β-tubulin from Sigma-Aldrich; and PDI/PDIA2 from Enzo Life Science. The secondary antibodies used include HRP-conjugated goat anti-mouse or rabbit IgG (Jackson Immuno-Research Laboratories) and Alexa Fluor 488- or Alexa Fluor 555-conjugated goat anti-mouse or rabbit IgG (Invitrogen).

##### Analysis of Protein Solubility and Disulfide Bonds in HMCs

Cells expressing WT and mutated IRBP were harvested by centrifugation at 1000 × *g* for 10 min and then lysed in PBS, pH 7.4, containing 0.5% Triton X-100 (Tx) and 1 × EDTA-free protease inhibitor cocktails (Roche Applied Science). The supernatant containing Tx-soluble proteins was collected by centrifugation at 100,000 × *g* for 20 min. The pellet containing Tx-insoluble proteins was washed twice with ice-cold PBS, resuspended in lysis buffer (PBS, 0.1% SDS, 0.5% Triton X-100, and 0.1% Tween 20), and sonicated. The Tx-soluble and Tx-insoluble fractions were treated with 0 or 50 mm DTT at 70 °C for 10 min. DTT promotes reduction of disulfide bonds in proteins.

##### Immunoblot Analysis

Cell lysates were prepared in lysis buffer containing or lacking 50 mm DTT. All protein samples were separated in an 8% or 12% polyacrylamide gel by electrophoresis under denaturing conditions including 0.1% SDS (SDS-PAGE) and were transferred onto an Immobilon-P membrane (Millipore). The membrane was incubated with a primary antibody and a secondary antibody as described previously (37). Protein band(s) reacted with the antibodies was visualized with the ECL-Plus Western blotting Detection Reagent and an ImageQuant LAS 4000 (GE Healthcare). Signal intensity of each band was qualified using the ImageQuant TL software.

##### Immunocytochemistry

Cells grown on glass coverslips were fixed with freshly prepared 4% paraformaldehyde in PBS, pH 7.2, for 20 min and permeabilized by incubating with 0.2% Tx in PBS for 15 min. After blocking with 10% goat serum in PBS for 1 h, the cells were incubated with a primary antibody overnight at 4 °C and then with Alexa Fluor 488- or 555-conjugated secondary antibody at room temperature for 1 h. Before and after incubating with antibodies the cells were washed with 0.1% Tween 20 in PBS three times. Nuclei were stained with 5 μg/ml DAPI (Sigma-Aldrich). Cells were mounted on glass slides, and images were captured with a Zeiss LSM 510 Meta confocal laser microscope.

##### Immunoprecipitation

Cells were harvested and lysed in a 50 mm Tris-HCl buffer, pH 7.4, containing 150 mm NaCl, 1 mm EDTA, 0.5% Nonidet P-40, and protease inhibitors. After centrifugation, the supernatants were collected, and immunoprecipitation was carried out with a Dynabeads Immunoprecipitation Kit (Invitrogen) and an affinity-purified antibody against IRBP or anti-FLAG M2 affinity gel (Sigma-Aldrich). Precipitated proteins were dissociated from the beads by elution with the SDS sample buffer and heated at 70 °C for 10 min. The proteins were separated in a 10% or 12% gel by SDS-PAGE followed by immunoblot analysis or Coomassie Brilliant Blue staining.

##### ER Stress-activated Indicator Assay

This assay was performed as described previously (38). Fluorescence images from 293T-LC cells co-transfected with pCAX-F-*XBP1*ΔDBD-*venus* and either pRK5 control, IRBP, or D1080N IRBP expression plasmid were superimposed on the phase-contrast images using the Zeiss confocal laser microscope. Fluorescence intensities in the transfected cell lysates in digitonin buffer (50 mm Tris-HCl, pH 7.5, 1 mm EDTA, 10 mm EGTA, and 10 μm digitonin) were measured using the Thermo Scientific Appliskan (emission at 535 nm; excitation at 485 nm). The fluorescence intensity of each sample was normalized to the β-galactosidase activity in the cell lysates.

##### MS Analysis

Protein identification by MS analysis was carried out as described previously (39). In brief, protein bands digested with trypsin were loaded into a Dionex PepMap C18 trap column and separated by a New Objective reversed phase C18 Picofrit column/emitter. Peptide mass was determined by a Thermo-Fisher LTQXL linear ion trap mass spectrometer coupled with an Eksigent nanoLC. The raw data were analyzed by the Mascot search engine (Matrix Science Inc.) against the human SwissProt database (false discovery rate <5%) to identify proteins for the bands.

##### Statistical Analysis

All analyses were performed using the SigmaPlot software version 11. Data are expressed as the mean ± S.D. Differences were evaluated using a one-way analysis of variance, and *p* < 0.01 was considered statistically significant.

## RESULTS

### 

#### 

##### The RP-associated Mutation Abolishes Secretion of IRBP

**FIGURE 1.**
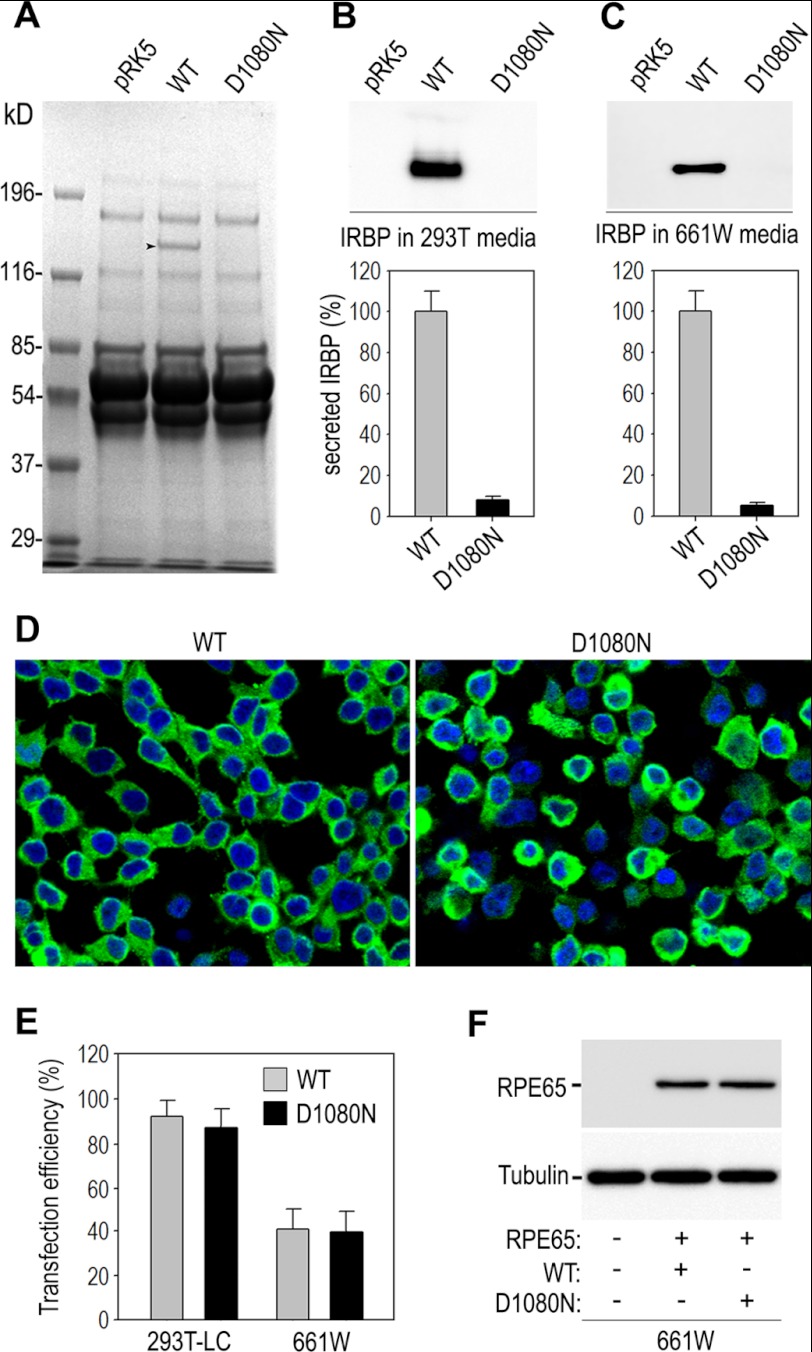
**D1080N mutation abolished secretion of IRBP.** *A*, proteins in the media of 293T-LC cells transfected with pRK5 mock vector or plasmid encoding wild-type (WT) or mutant (D1080N) IRBP were separated in an 8% gel and stained with Coomassie Brilliant Blue. *Arrowhead* indicates a protein band of ∼140 kDa that is present only in the medium of cells transfected with WT IRBP. *B* and *C*, representative immunoblot analysis shows the presence of WT IRBP, but not D1080N IRBP, in the media of 293T-LC (*B*) and 661W (*C*) cells transfected with the indicated plasmids. Relative content of WT and D1080N IRBP secreted into the media was quantified using an ImageQuant TL and expressed in histograms as percentage of WT IRBP. *Error bars* show S.D. (*n* = 3). *D*, confocal microscopic images of immunocytochemistry show expression of WT and mutant IRBP (*green*) in the transfected 293T-LC cells. Cell nuclei were stained with DAPI. *E*, transfection efficiencies of WT and mutant IRBP were calculated from the ratio of IRBP-positive cells to DAPI-positive cells in triplicate transfections. *F*, immunoblot analysis shows similar expression levels of RPE65 co-transfected with WT or mutant IRBP in 661W cells.

Because IRBP is a secretory protein, we tested whether the D1080N mutation affects secretion of IRBP. We expressed wild-type and mutant IRBPs in 293T-LC cells by transient transfection. Proteins in media of the transfected cells were analyzed by Coomassie Brilliant Blue staining and immunoblot analysis. Coomassie Brilliant Blue staining showed that only the medium of cells transfected with wild-type IRBP-expressing plasmid contained an ∼140-kDa protein (Fig. 1*A*), which is similar to the predicted molecular mass of IRBP. This 140-kDa protein was not detectable in either medium of cells transfected with the pRK5 mock vector or the plasmid encoding D1080N IRBP (Fig. 1*A*). Consistent with this result, immunoblot analysis using an IRBP antibody detected an abundant amount of a 140-kDa protein in the medium of cells transfected with wild-type IRBP-expressing plasmid (Fig. 1*B*). The medium of cells transfected with D1080N IRBP-expressing plasmid contained a trace or undetectable amount of IRBP (Fig. 1*B*). To confirm this result, we performed a similar experiment in 661W cells, which express cone photoreceptor-specific proteins (35). Immunoblot analysis showed that the only medium of cells transfected with wild-type IRBP-expression plasmid contained a significant amount of IRBP (Fig. 1*C*). Quantitative immunoblot analysis indicated that the relative amounts of D1080N IRBP secreted into the medium by 293T-LC and 661W cells were ∼8 and 5% of the secreted wild-type IRBP (Fig. 1, *B* and *C*), respectively. To confirm that this lower content of the mutant IRBP in the medium was not due to lower transfection efficiency of mutant IRBP we performed immunocytochemistry using an antibody against IRBP. As shown in Fig. 1*D*, a majority of 293T-LC cells expressed wild-type or mutant IRBP transfected, whereas nontransfected cells did not express any IRBP (data not shown). The ratio of IRBP-positive cells to DAPI-positive cells indicated that ∼90% of 293T-LC cells and 40% of 661W cells were transfected with wild-type or mutant IRBP (Fig. 1*E*). In addition, expression levels of RPE65 co-transfected with D1080N IRBP in 661W cells were similar to those in the cells co-transfected with wild-type IRBP (Fig. 1*F*), suggesting that transfection and expression efficiencies of transfected genes were similar in wild-type *versus* mutant IRBP-transfected cells. Taken together, the results described above indicate that the RP-associated mutation abolished secretion of IRBP, which leads to loss of normal function of IRBP.

##### D1080N IRBP Forms Insoluble HMCs via Disulfide Bonds

**FIGURE 2.**
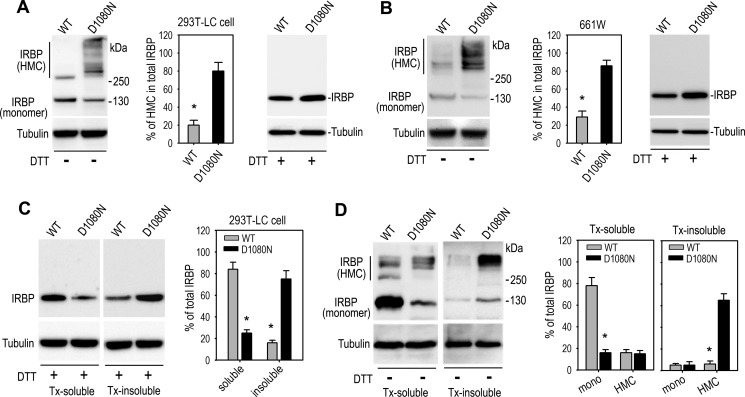
**D1080N mutation resulted in formation of insoluble HMCs via disulfide bonds within the cells.** *A* and *B*, the 293T-LC (*A*) and 661W (*B*) cells expressing WT or D1080N IRBP were evaluated by immunoblot analysis in the presence or absence of reducing reagent DTT. Monomer and HMCs of IRBP are indicated at the *side* of each immunoblot. *Numbers* on the *right side* of the immunoblots indicate kDa of molecular mass markers. Relative content of HMCs is shown in histograms as a percentage of total IRBP. *C*, immunoblot analyses performed in the presence of DTT show abundant distribution of D1080N IRBP in the Tx-insoluble cellular fraction and WT IRBP in the Tx-soluble fraction of 293T-LC cells. *D*, immunoblot analyses performed in the absence of DTT show abundant distribution of mutant IRBP HMCs in the Tx-insoluble cellular fraction and monomeric WT IRBP in the Tx-soluble fraction of 293T-LC cells. *Asterisks* in all histograms indicate significant differences between WT and mutant IRBPs (*p* < 0.001). *Error bars* show S.D. (*n* = 3).

As the first step to define the molecular mechanisms by which the mutation abolishes secretion of IRBP, we analyzed the transfected 293T-LC and 661W cells by immunoblot analysis in the absence of reducing reagent. We observed that ∼70–80% of wild-type IRBP appeared to be monomeric IRBP as it migrated to an approximate 140-kDa position in the gel (Fig. 2, *A* and *B*). In contrast, at least 70% of D1080N IRBP migrated much slower compared with monomeric IRBP (Fig. 2, *A* and *B*), suggesting that the mutated IRBP formed HMCs in both 293T-LC and 661W cells. To test whether protein disulfide bonds were involved in formation of the HMCs, we treated the cell lysates with DTT that cleaves protein disulfide bonds. Immunoblot analysis showed that the amount of the HMC decreased whereas the amount of monomeric IRBP increased by the DTT treatment (Fig. 2, *A* and *B*), suggesting that the HMC was formed by protein disulfide bonds.

Because abnormal disulfide bond formation has often been observed in mutated secretory proteins that form insoluble protein aggregates (40, 41), we analyzed the solubility of D1080N IRBP in the presence of 0.5% Tx and 50 mm DTT. As shown in Fig. 2*C*, 75% of wild-type IRBP was included in the Tx-soluble fraction, whereas 80% of D1080N IRBP was contained in the Tx-insoluble cellular fraction, suggesting that the RP-associated IRBP formed insoluble aggregates.

Because a majority of D1080N formed HMCs, we tested whether the Tx insolubility of D1080N IRBP was due to the formation of HMCs. We prepared Tx-soluble and -insoluble fractions in the absence of DTT and separated the fractions by SDS-PAGE without reducing reagent. Immunoblot analysis showed that at least 80% of total Tx-insoluble D1080N IRBP was HMCs (Fig. 2*D*), indicating that D1080N IRBP is prone to form insoluble HMCs. In contrast, most of the soluble IRBP was monomeric wild-type IRBP (Fig. 2*D*).

##### PDIA2, Cys-304, and Cys-1175 Are Important for Forming Nonsecretory D1080N IRBP

**FIGURE 3.**
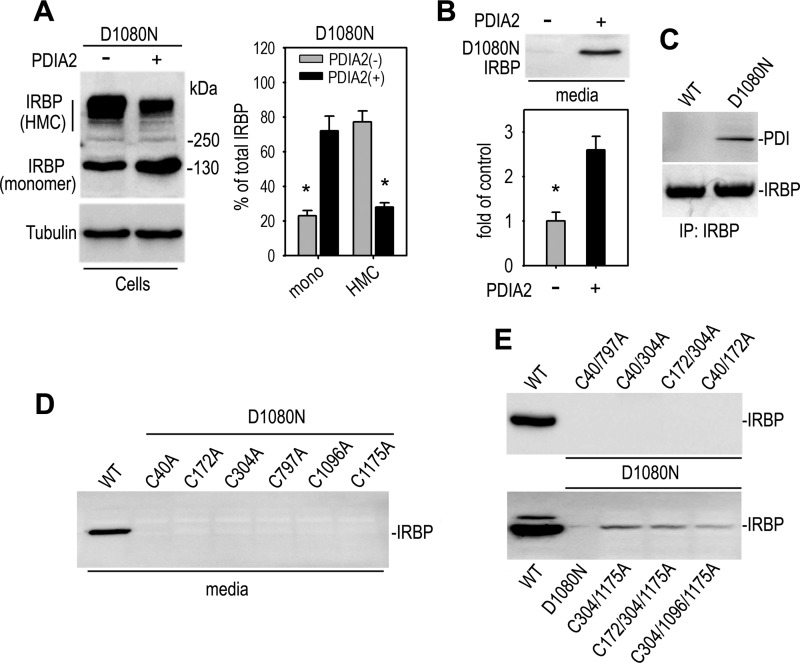
**PDIA2, Cys-304, and Cys-1175 are important for formation of nonsecretory D1080N IRBP.** *A* and *B*, PDIA2 inhibited formation of HMC (*A*) and promoted secretion (*B*) of D1080N IRBP in the co-transfected 293T-LC cells. Relative contents of monomer (*mono*) and HMCs of D1080N IRBP in the cells co-transfected or not co-transfected with PDIA2 are shown in the histogram as percentage of total IRBP (*A*). The histogram in *B* shows relative content of D1080N IRBP in media of the cells co-transfected or not co-transfected with PDIA2. *C*, interaction of PDIA2 with D1080N IRBP is shown. Cells expressing WT or D1080N IRBP were immunoprecipitated with an IRBP antibody, and the precipitates were probed with antibodies against PDIA2 or IRBP. *D* and *E*, effects of single (*D*) and double or triple (*E*) Cys-to-Ala substitutions at the indicated positions on secretion of D1080N IRBP are shown. Secretion of the mutated IRBP was determined by immunoblot analysis of IRBP in media of the transfected cells. Note significant secretion of D1080N IRBP with double Cys-to-Ala substitution at positions 304 and 1175 (C304A/C1175A).

PDIA2, a member of PDI family, is an ER protein chaperone that catalyzes the formation and breakage of disulfide bonds between cysteine residues and can inhibit aggregation of misfolded proteins by catalyzing rearrangement of -S-S- bonds in proteins (42). We therefore co-expressed PDIA2 and D1080N IRBP in 293T-LC cells. As shown in Fig. 3*A*, the HMC was significantly reduced, and secretion of D1080N IRBP was partially rescued by co-expression of PDIA2. Immunoprecipitation confirmed that PDIA2 interacted preferentially with D1080N IRBP (Fig. 3*B*). These results suggest that abnormal formation of disulfide bond(s) is involved in the molecular mechanisms underlying formation of the nonsecretory IRBP.

To identify the Cys residue(s) involved in the formation of nonsecretory mutant IRBP, we systematically changed six conserved Cys residues at positions 40, 172, 304, 797, 1096, and 1175 to Ala in D1080N IRBP. Immunoblot analysis for IRBP in media of the transfected cells showed that single Cys-to-Ala substitutions did not alter secretion of D1080N IRBP (Fig. 3*D*). We then introduced double and triple Cys-to-Ala substitutions into D1080N IRBP. Among the mutated proteins, D1080N IRBP with double Cys-to-Ala substitutions at positions 304 and 1175 was significantly secreted into the media from the transfected cells (Fig. 3*E*), suggesting that these two Cys residues are critical for forming nonsecretory HMCs of the RP-associated IRBP.

##### The RP-associated IRBP Fails to Transport to the Golgi and Causes ER Stress

**FIGURE 4.**
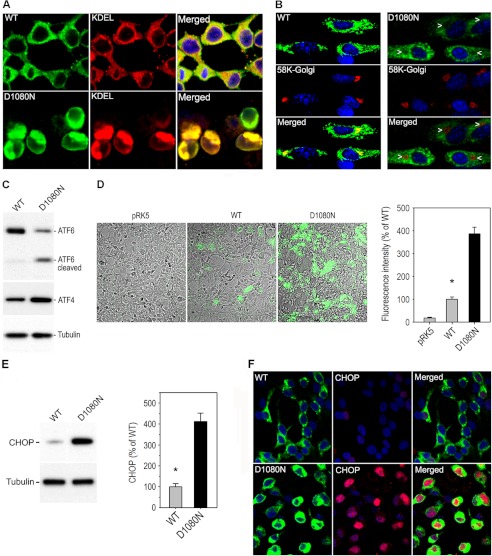
**D1080N IRBP accumulated in the ER and caused ER stress.** *A*, immunocytochemistry of transfected 293T-LC cells shows localization of both WT and D1080N IRBPs on the ER labeled with an anti-KDEL antibody, an ER marker. Note the intense signal of D1080N IRBP in the ER. *B*, immunocytochemistry shows co-localization of Golgi marker with WT IRBP, but not D1080N IRBP, in 661W cells. *Arrowheads* point to the places where D1080N IRBP is negative but Golgi marker is positive. *C*, immunoblot analysis shows increased content of ATF4 and active form of ATF6 in cells expressing D1080N IRBP. *D*, fluorescence microscopy shows distinct expression levels of XBP1-Venus fusion protein (*green*), an ER stress-activated indicator, in 293T-LC cells transfected with pRK5 mock or plasmid encoding WT or D1080N IRBP. These fluorescence images are superimposed on the phase-contrast images. *E*, immunoblot analysis shows up-regulation of CHOP in cells expressing D1080N IRBP. Relative contents of CHOP in cells expressing WT or D1080N IRBP are shown in the histogram. *Error bars* represent S.D. (*n* = 3); *asterisk* denotes a significant difference (*p* < 0.001). *F*, immunocytochemistry for CHOP in the cells expressing WT or D1080N IRBP is shown.

Based on the results described above, we predicted that D1080N IRBP failed to form the native structure of IRBP and, therefore, cannot be transported to the Golgi apparatus from the ER. To test this hypothesis, we compared subcellular localization of wild-type and mutant IRBPs in 293T-LC and 661W cells. Confocal microscopy images showed that wild-type IRBP was co-localized with both ER and Golgi makers (Fig. 4, *A* and *B*), indicating that wild-type IRBP is able to translocate to the Golgi from the ER for its secretion. In contrast, we observed that D1080N IRBP was not co-localized with the Golgi marker (Fig. 4*B*). Instead, D1080N IRBP was so densely localized in the ER that the ER shape stained with both D1080N IRBP and the ER marker appears abnormal in 293T-LC cells (Fig. 4*A*, *bottom panel*). These observations suggest that the RP-associated IRBP, which cannot be transported to the Golgi, accumulated in the ER.

Accumulation of misfolded protein in the ER induces the UPR and ER stress. We therefore tested whether the UPR is activated in cells expressing D1080N IRBP. As shown in Fig. 4*C*, expression levels of ATF4 and the active form of cleaved ATF6 were significantly increased in cells expressing D1080N IRBP, suggesting that D1080N IRBP triggered the UPR. To confirm this result, we co-transfected pCAX-F-*XBP1*ΔDBD-*venus*, an ER stress-activated indicator (38), into 293T-LC cells with pRK5, IRBP, or D1080N IRBP expression plasmid. The pCAX-F-*XBP1*ΔDBD-*venus* expresses XBP1-Venus fusion fluorescent protein only when its mRNA is spliced by inositol-requiring kinase 1/endoribonuclease (IRE1) in the cells experiencing ER stress. As shown in Fig. 4*D*, cells co-transfected with D1080N IRBP expressed a significantly higher level of the fusion fluorescent protein compared with the pRK5- or wild-type IRBP-co-transfected cells. Consistent with these results, immunoblot analysis showed that CHOP, a key transcription factor involved in ER stress-induced apoptosis (34), was up-regulated ∼4-fold in cells expressing D1080N IRBP compared with cells expressing wild-type IRBP (Fig. 4*E*). In addition, immunocytochemistry indicated that the up-regulated CHOP was translocated into the nuclei of cells expressing D1080N IRBP (Fig. 4*F*).

##### D1080N IRBP Binds Protein Chaperones and Is Degraded by ERAD

**FIGURE 5.**
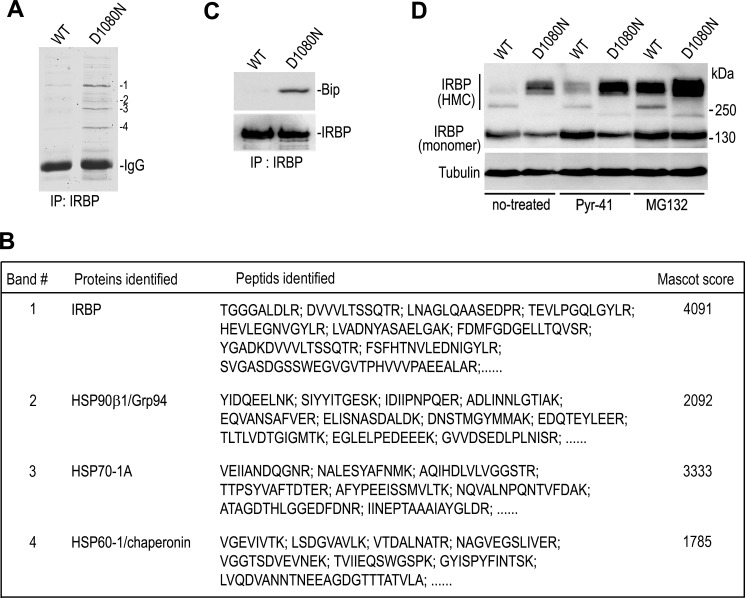
**D1080N IRBP bound with protein chaperones undergoes ER-associated degradation.** *A*, proteins bound to IRBP in 293T-LC cells transfected with WT or D1080N IRBP expression plasmid were co-immunoprecipitated (*IP*) with an IRBP antibody and stained with Coomassie Brilliant Blue after separating in a 10% polyacrylamide gel. *B*, LC-MS identification of protein bands marked in *A* is shown. *C*, interaction of D1080N IRBP with ER chaperone protein is shown. The 293T-LC cells expressing WT or D1080N IRBP were immunoprecipitated with an antibody against IRBP, and the precipitates were probed with an antibody against BiP. The membranes were reprobed with an IRBP antibody. *D*, immunoblot of the cells expressing WT or mutant IRBP were analyzed. The cells were treated with E1 inhibitor (PYR-41) or proteasome inhibitor (MG132) following transfection.

To identify protein(s) that interacts with D1080N IRBP, we did an immunoprecipitation using an antibody against IRBP and the cells expressing wild-type or D1080N IRBP. Coomassie Brilliant Blue staining of the immunoprecipitated proteins showed that several proteins strongly interacted with D1080N IRBP (Fig. 5*A*). Through mass spectrometry analysis, we identified heat shock proteins (HSPs), such as HSP-90β1/94-kDa glucose-regulated protein (Grp94), HSP70-1A and HSP60/chaperonin as the proteins that interact preferentially with the RP-associated IRBP (Fig. 5*B*). HSPs are protein chaperones that interact with misfolded proteins (43). Because D1080N IRBP was misfolded and accumulated in the ER, we hypothesize that the mutant IRBP may interact with ER-resident chaperone proteins such as PDI/PDIA2 and binding immunoglobulin protein (BiP). To test this hypothesis, we analyzed the immunoprecipitated fractions with antibodies against PDI or Bip. As predicted, immunoblot analysis showed that PDI/PDIA2 and BiP preferentially bound to D1080N IRBP (Figs. 3*C* and 5C). Taken together, our observation suggests that the mutant IRBP was misfolded and bound to chaperone proteins.

Interaction between misfolded proteins and chaperone proteins facilitates elimination of misfolded proteins via the ERAD pathway (44), which is a cellular adaptive response. To test whether D1080N IRBP enters the ERAD pathway, we treated cells expressing wild-type or D1080N IRBP with MG132, an inhibitor of the proteasome, or PYR-41, an inhibitor of E1 ubiquitin-activating enzyme (45). Immunoblot analysis of the cells showed that D1080N IRBP, including the HMC and monomeric form, increased markedly in MG132-treated cells compared with untreated cells (Fig. 5*D*), suggesting that some of the D1080N IRBP was degraded by proteasomes. The HMC and monomeric forms of the mutant IRBP increased substantially when cells were treated with the E1 inhibitor whereas wild-type IRBP was only modestly affected by the inhibitor (Fig. 5*D*), indicating that a portion of D1080N IRBP was preferentially degraded via the ubiquitin-proteasome pathway.

##### D1080N IRBP Inhibits Secretion of Wild-type IRBP by IRBP Homophilic Interaction

**FIGURE 6.**
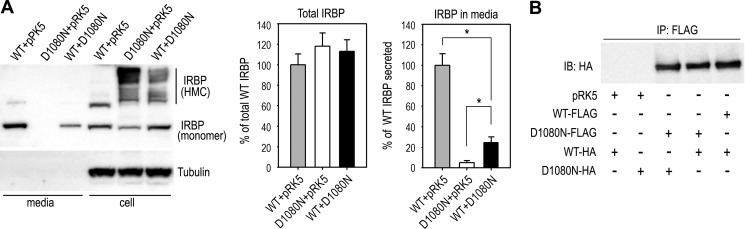
**D1080N IRBP inhibited secretion of WT IRBP by direct interaction.** *A*, immunoblot analysis for IRBP in media and lysates of 293T-LC cells transfected with the indicated combination of plasmids. Relative contents of total IRBP in media and cells were determined by measuring intensities of IRBP bands and are expressed in the histogram (*middle panel*) as percentage of WT IRBP in pRK5 co-transfected samples. The histogram in the *right panel* shows relative content of IRBP secreted into media of the transfected cells. *Error bars* represent S.D. of three independent experiments; *asterisks* denote significant differences between the indicated groups (*p* < 0.001). *B*, interactions between WT IRBPs, D1080N IRBPs, and between WT and D1080B IRBPs. Cells expressing WT and D1080N IRBPs tagged with FLAG or HA epitope were immunoprecipitated with a FLAG antibody. The precipitates were then probed with an HA antibody.

To test whether D1080N IRBP interferes with secretion of wild-type IRBP, we determined the relative amount of IRBP in the media of cells co-transfected with wild-type IRBP plasmid plus pRK5 or D1080N IRBP plasmid. Quantitative immunoblot analysis of IRBP showed that total expression levels of IRBP (including cellular and secreted IRBP) were similar in both groups of transfected cells (Fig. 6*A*). However, the amount of IRBP secreted into the medium of cells expressing wild-type and D1080N IRBPs was only 30% of cells expressing wild-type IRBP alone (Fig. 6*A*). These results indicate that D1080N IRBP inhibits secretion of wild-type IRBP. One of the possible explanations for this result is that the nonsecretory mutant IRBP accumulated in the ER (Fig. 4*A*) trapped wild-type IRBP by physical interaction. To test this possibility we co-expressed wild-type and D1080N IRBPs fused with the FLAG or HA epitope. Following immunoprecipitation with an antibody against the FLAG epitope, we detected IRBP in the precipitates with an antibody against the HA epitope. The results shown in Fig. 6*B* indicate that D1080N IRBP bound not only the mutant IRBP but also wild-type IRBP, suggesting that one of the mechanisms by which D1080N IRBP inhibits secretion of wild-type IRBP is by direct interaction between wild-type IRBP and the nonsecretory D1080N IRBP accumulated in the ER.

##### Chemical Chaperones and Low Temperature Partially Rescue Secretion of D1080N IRBP

**FIGURE 7.**
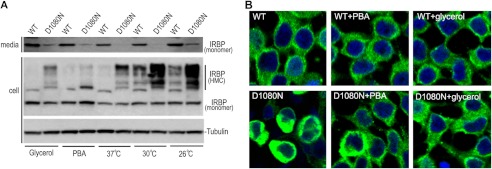
**Low temperature and chemical chaperones promote secretion of D1080N IRBP.** *A*, immunoblot analyses of media (*top panel*) and lysates (*middle and bottom panels*) of 661W cell maintained in the presence of 5% glycerol, 4 mm PBA, or at the indicated temperature following transfection of WT and D1080N IRBP expression plasmid. *B*, confocal microscopy of the 293T-LC cells maintained in the presence or absence of PBA or glycerol. Note morphological recovery of cells expressing D1080N IRBP following chemical treatment.

Previous studies have shown that low temperature and chemical chaperones can help proper folding of many mutated proteins (28, 40, 46, 47). We therefore tested whether PBA, glycerol, and low temperature can rescue secretion of the mutated IRBP. We incubated 661W cells expressing wild-type or D1080N IRBP at different temperatures (37, 30, and 26 °C) in the presence or absence of 4 mm PBA or 5% glycerol. Immunoblot analysis of media from the cells showed that the mutant IRBP was significantly secreted into the media of cells maintained at 26 °C and treated with PBA or glycerol (Fig. 7*A*, *top panel*). The increased secretion by the chemical treatments was accompanied by a significant reduction in HMC formation of D1080N IRBP in the cells (Fig. 7*A*), whereas incubation of cells at low temperature increased the formation of HMCs (Fig. 7*A*). This increased formation of HMCs was most likely due to reduction of proteasome activity at 26 °C. The rescue of the mutant IRBP secretion was also confirmed in 293T-LC cells (data not shown). In 293T-LC cells, we also observed that the abnormal cell morphology caused by accumulation of the mutant IRBP in the ER (Figs. 4*A* and 7*B*) was significantly rescued by the treatment with chemical chaperones (Fig. 7*B*). Similar to wild-type IRBP, D1080N IRBP was diffusely distributed in the cells treated with PBA (Fig. 7*B*), suggesting that the rescue effect of the chemical chaperones was most likely due to improved folding of D1080N IRBP.

## DISCUSSION

In the present study, we analyzed the molecular pathogenic basis for an RP-associated mutation in the human *IRBP* gene. Our results suggest two possible disease mechanisms linking the D1080N mutation with RP: (i) loss of normal function due to failure of secretion and (ii) gain of cytotoxic function causing ER stress.

IRBP is a secretory protein and is the most abundant soluble protein component of the interphotoreceptor matrix (48). Previous studies on *Irbp*^−^*^/^*^−^ mice have shown that IRBP plays pivotal roles in rod and cone survival (13, 18–20) and cone visual function (13, 14, 49). Its neonatal expression and secretion patterns in wild-type, retinal degeneration (*rd*), and retinal degeneration slow (*rds*) retinas suggest that it plays its role after it is secreted into the interphotoreceptor matrix (50). In the present study, we observed that wild-type IRBP was abundantly secreted from both photoreceptor-derived and kidney-derived cell lines (Fig. 1, *A–C*). In contrast, D1080N IRBP was not or rarely secreted from the same cell lines (Fig. 1,*A–C*) although the mutant IRBP was otherwise abundantly expressed at levels comparable with wild-type IRBP (Figs. 1*D* and 2, *A* and *B*). The secretory defect of D1080N IRBP was corroborated by its subcellular localization in the cells (Fig. 4, *A* and *B*). Wild-type IRBP was localized in both the ER and the Golgi apparatus (Fig. 4, *A* and *B*). However, D1080N IRBP was not localized in the Golgi (Fig. 4*B*), but densely accumulated in the ER (Fig. 4*A*), suggesting that the mutant IRBP was not competent for transport to the Golgi from the ER.

The molecular basis for the secretory defect of D1080N IRBP seems to be abnormal formation of protein disulfide bonds caused by misfolding of IRBP due to the mutation. The abnormal disulfide bonds are formed, most likely, between IRBP and other proteins because the HMCs contained several HMCs with distinct molecular masses (Fig. 2), and some of them are clearly different from dimeric, trimeric, and tetrameric IRBP (Fig. 2, *A* and *B*). D1080N IRBP physically interacts with distinct heat shock proteins (Fig. 5, *A* and *B*) and ER chaperone proteins (Figs. 3*C* and 5*C*). Because D1080N IRBP exhibited homophilic interactions (Fig. 6*B*) we cannot rule out the possibility of homotypic formation of disulfide bonds between mutant IRBPs. Although a small portion of wild-type IRBP also formed HMCs (Fig. 2, *A* and *B*), the amount of HMC-containing wild-type IRBP was significantly smaller than that of the mutant IRBP in both 293T-LC and 661W cells (Fig. 2, *A* and *B*).

The majority of D1080N IRBP was insoluble in the solution containing Triton X-100 whereas the majority of wild-type IRBP was soluble in the same solution (Fig. 2*D*). The Triton X-100 detergent solubility characteristics of mutant and normal proteins have been used to determine the aggregation state of misfolded mutant proteins (51). The insolubility of D1080N IRBP in the detergent-containing solution suggests that the mutant IRBP is prone to form aggregates due to misfolding. In support of this possibility, D1080N IRBP was densely accumulated in the ER (Figs. 1*D* and 4*A*) and preferentially interacted with chaperone proteins (Figs. 3*C* and 5*C*), which have been shown to bind many misfolded proteins (32).

PDIA2 is a member of the PDI family that functions as an isomerase to catalyze thiol-disulfide exchange of Cys residues and as a chaperone to assist protein folding. Overexpression of PDIA2 reduced HMC formation of D1080N IRBP and promoted secretion of the mutant IRBP (Fig. 3, *A* and *B*), confirming that the HMC production of D1080N IRBP involves formation of abnormal disulfide bonds which can be modulated by PDIA2. Overexpression of PDI has been documented to protect neurons from neurodegenerative disease by decreasing aggregation of mutant proteins (42), although under certain conditions PDI facilitates aggregation of proteins (52). Primm *et al.* proposed a working model that accounts for the ability of PDI to stimulate protein aggregation at low concentrations but inhibit the aggregation at high concentrations (53). Recent studies confirmed that at lower concentration, PDI facilitated aggregation of unfolded proteins and exhibited antichaperone activity. At high concentrations, however, PDI acted as a protector against aggregation of protein (54). These studies and our observation suggest that PDI plays an important role in proper folding of IRBP by regulating formation of disulfide bond between Cys residues in IRBP.

Human IRBP has six Cys residues that are conserved in mammalian IRBPs. Single substitution of these Cys to Ala did not affect secretion of the mutant IRBPs. However, double Cys-to-Ala substitutions at positions 304 and 1175 significantly increased secretion of the RP-associated IRBP (Fig. 3*E*), suggesting that these two Cys residues are critical for forming nonsecretory HMCs of D1080N IRBP. Determination and further studies of IRBP structure are needed for understanding the molecular mechanism by which the two Cys residues regulate folding of D1080N IRBP.

The ER is the site of synthesis and folding of secretory proteins. Both constitutive secretion and regulated secretion require transportation of proteins synthesized in the ER to the *cis*-Golgi and then to the *trans*-Golgi. In general, only proteins that have correct folding and post-translational modifications can be transported to the Golgi apparatus. Because D1080N IRBP formed insoluble HMCs due to misfolding, transport to the Golgi failed. Instead, the misfolded mutant IRBP densely accumulated in the ER (Fig. 4*A*). In contrast, wild-type IRBP readily transported to the Golgi (Fig. 4*B*).

When misfolded protein accumulates in the ER, cells initiate the UPR to maintain ER homeostasis and function. ATF6, an ER-located transmembrane protein, is a key player in activation of the UPR. Upon sensing accumulation of misfolded protein, ATF6 is processed in a Site 1 and Site 2 protease-dependent manner (24). Its cytoplasmic domain enters the nucleus and promotes transcription of chaperone proteins. In the D1080N IRBP-expressing cells, ATF6 was significantly cleaved (Fig. 4*C*), suggesting that it was activated. The activated ATF6 induces transcription of XBP1 mRNA. IRE1, an ER protein that initiates UPR upon sensing accumulation of misfolded protein, splices out a 26-nucleotide from XBP1 mRNA to produce active XBP1 (25). To monitor activation of IRE1, we used the *XBP1-venus* fusion construct, a new ER stress-activated indicator that produces XBP1-Venus fusion green fluorescent protein only when IRE1 is activated (38). The fusion fluorescent protein was strongly expressed in the cells co-transfected with D1080N IRBP expression plasmid (Fig. 4*D*), suggesting that IRE1 was activated by the mutant IRBP. Taken together, these results indicate that the RP-associated IRBP activated UPR and caused ER stress.

CHOP, also known as growth arrest- and DNA damage-inducible gene 153 (55), is one of the critical downstream components of the UPR implicated in apoptosis in response to ER stress (34). CHOP is ubiquitously expressed at low levels in various cells and is present in the cytosol under nonstress condition (55). Various ER stresses and ATF4, which was up-regulated in cells expressing D1080N IRBP (Fig. 4*C*), have been shown to activate transcription of CHOP and its accumulation in the nucleus (23, 34, 55). Consistent with these studies, we observed that CHOP was robustly up-regulated and translocated to the nucleus in cells expressing D1080N IRBP (Fig. 4, *E* and *F*). CHOP regulates expression of genes involved in cell survival and death and thereby controls cell death under conditions associated with ER stress (34).

During short term expression of D1080N IRBP in 661W and 293T cells, no significant cell death was observed. A possible explanation for this is that an adaptive response to the ER stress is triggered. When proteins are misfolded, the ERAD pathway, which is a cellular protein quality control system, functions to address the situation. Binding of protein chaperones assists in proper folding and degradation of misfolded proteins (56, 57). In the present study, we observed that D1080N IRBP preferentially bound to ER chaperone proteins such as BiP and PDI as well as other heat shock protein chaperones (Figs. 3*C* and 5, *A–C*) and that at least some mutant IRBP was degraded by the ubiquitin-proteasome system (Fig. 5*D*). These responses might protect cells from serious damage caused by accumulation of misfolded mutant IRBP. However, as is the case for a wide variety of retinal degenerative diseases associated with protein misfolding and aggregation (27, 29, 31, 33, 58), long term retention of misfolded insoluble mutant IRBP in the ER may impair the ubiquitin-proteasome system, cause ER stress, and eventually activate apoptotic pathways in photoreceptors.

RP caused by the D1080N mutation is an autosomal recessive form of RP (2). In our experiments, which mimicked the heterozygous condition (co-expression of wild-type and mutant IRBPs), the mutated IRBP strongly inhibited secretion of wild-type IRBP (Fig. 6*A*). Because the mutant IRBP accumulated in the ER and caused ER stress, the reduced secretion of wild-type IRBP might be due to the ER stress. Another possible mechanism by which D1080N IRBP reduces secretion of wild-type IRBP could be through direct interaction of wild-type IRBP with the nonsecretory mutant IRBP in the ER (Figs. 4*A* and 6*B*). In either case, D1080N IRBP may reduce normal function of IRBP by inhibiting secretion of wild-type IRBP. Because RP is a type of progressive retinal disease and D1080N IRBP causes ER stress in the cells, whether heterozygous carriers have any slowly progressed defect in retinal function or structure needs further studies.

Formation of cytotoxic aggregates is a common pathologic mechanism for numerous neurodegenerative diseases. Evidence is mounting that retinal degeneration-associated mutations in rhodopsin (58), EFEMP1 (27), ABCA4 (29), carbonic anhydrase IV (31), and cyclic nucleotide-gated channels (33) lead to the production of misfolded proteins which induces ER stress and subsequent photoreceptor death. Such protein misfolding can be rescued by low temperature and chemical chaperones (28, 40, 46, 47). Incubation of cells expressing D1080N IRBP at 26 °C promoted secretion of the mutant IRBP (Fig. 7*A*). Moreover, treatment of cells with PBA and glycerol significantly rescued secretion and reduced aggregation of the misfolded IRBP (Fig. 7, *A* and *B*). PBA is a US Food and Drug Administration-approved safe oral medication (the trade name is Buphenyl® registered in the United States and Ammonaps® in Europe). Our findings suggest that PBA medication may be a promising candidate for the development of therapeutic intervention for preventing or delaying vision loss and retinal degeneration in patients with the RP-associated IRBP mutation.

## Footnotes

4 The abbreviations used are:

RP: retinitis pigmentosa
ATF: activating transcription factor
BiP: binding immunoglobulin protein
CHOP: C/EBP homologous protein
ER: endoplasmic reticulum
ERAD: ER-associated degradation
HMC: high molecular weight complex
IRBP: interphotoreceptor retinoid-binding protein
IRE1: inositol-requiring kinase 1/endoribonuclease
PBA: 4-phenylbutyrate
PDI: protein disulfide isomerase
RPE: retinal pigment epithelium
Tx: Triton X-100
UPR: unfolded protein response
XBP1: X-box-binding protein-1.
